# A Rare Instance of Simultaneous Infection with Disseminated Nocardia and Pulmonary Aspergillus in a Patient Receiving Treatment with Ibrutinib for Chronic Lymphocytic Leukemia

**DOI:** 10.7759/cureus.3427

**Published:** 2018-10-08

**Authors:** Jennifer Dotson, Heather Katz, Hassaan Jafri, Muhammad Omer Jamil, Kara S Willenburg

**Affiliations:** 1 Oncology, Joan C. Edwards School of Medicine - Marshall University, Huntington, USA; 2 Hematology / Oncology, Joan C. Edwards School of Medicine - Marshall University, Huntington, USA; 3 Internal Medicine, Joan C. Edwards School of Medicine - Marshall University, Huntington, USA; 4 Hematology / Oncology, Joan C. Edwards School of Medicine - Marshall University, Huntington , USA; 5 Infectious Disease, Joan C. Edwards School of Medicine - Marshall University, Huntington, USA

**Keywords:** chronic lymphocytic leukemia, ibrutinib, nocardia

## Abstract

Disseminated Nocardia infections are a rare occurrence that typically occur in immunocompromised hosts. Chronic lymphocytic leukemia (CLL) is a hematologic malignancy which makes patients susceptible to infections through various mechanisms. The treatments for CLL also target immunologic pathways which can contribute to infections. Ibrutinib is a commonly utilized tyrosine kinase inhibitor for CLL which targets the Bruton tyrosine kinase pathway. Although it is generally well tolerated, patients sometimes experience infections while on this medication, however, opportunistic infections are unusual. We present a rare, fatal case of a patient who developed simultaneous infections with bronchopulmonary *Aspergillus* and disseminated Nocardiosis to the subcutaneous tissues, lymph nodes and central nervous system while on treatment with ibrutinib for CLL.

## Introduction

Disseminated *Nocardia* infections are usually seen in immunocompromised patients, such as those patients with acquired immunodeficiency syndrome (AIDS) or on chronic steroids or immunosuppressants. Chronic lymphocytic leukemia (CLL) is a hematologic malignancy that predisposes patients to infections, especially encapsulated organisms [1]. Infections from opportunistic organisms are less common. CLL is an abnormal proliferation of mature monoclonal B-lymphocytes which impacts the immune system in a multitude of ways, both from the disease itself as well as the consequences of treatment [1]. Because of this, infections are a major cause of morbidity in these patients. Ibrutinib is an oral medication used in the treatment of CLL that works by irreversible inhibition of Bruton tyrosine kinase, which is involved in the B-cell antigen receptor pathway [2,3]. Infections have been reported in up to 24% of patients who receive Ibrutinib, including pneumonia and more uncommon infections such as *Aspergillus *[4]. We report a rare fatal instance of a patient with CLL on ibrutinib who contracted fatal simultaneous infections with bronchopulmonary *Aspergillus* and disseminated *Nocardia* to the subcutaneous tissues, bones, lymph nodes and central nervous system (CNS).

## Case presentation

A 64-year-old female with a past medical history of CLL being treated with ibrutinib, presented with fever, chills, night sweats, productive cough and lower back pain. She had been diagnosed with CLL (Rai stage I) five years prior, along with 17p deletion, and was initially treated with bendamustine and rituximab, with an excellent response. Two years later, she became symptomatic with generalized lymphadenopathy and was started on ibrutinib 420 mg daily.

**Figure 1:**
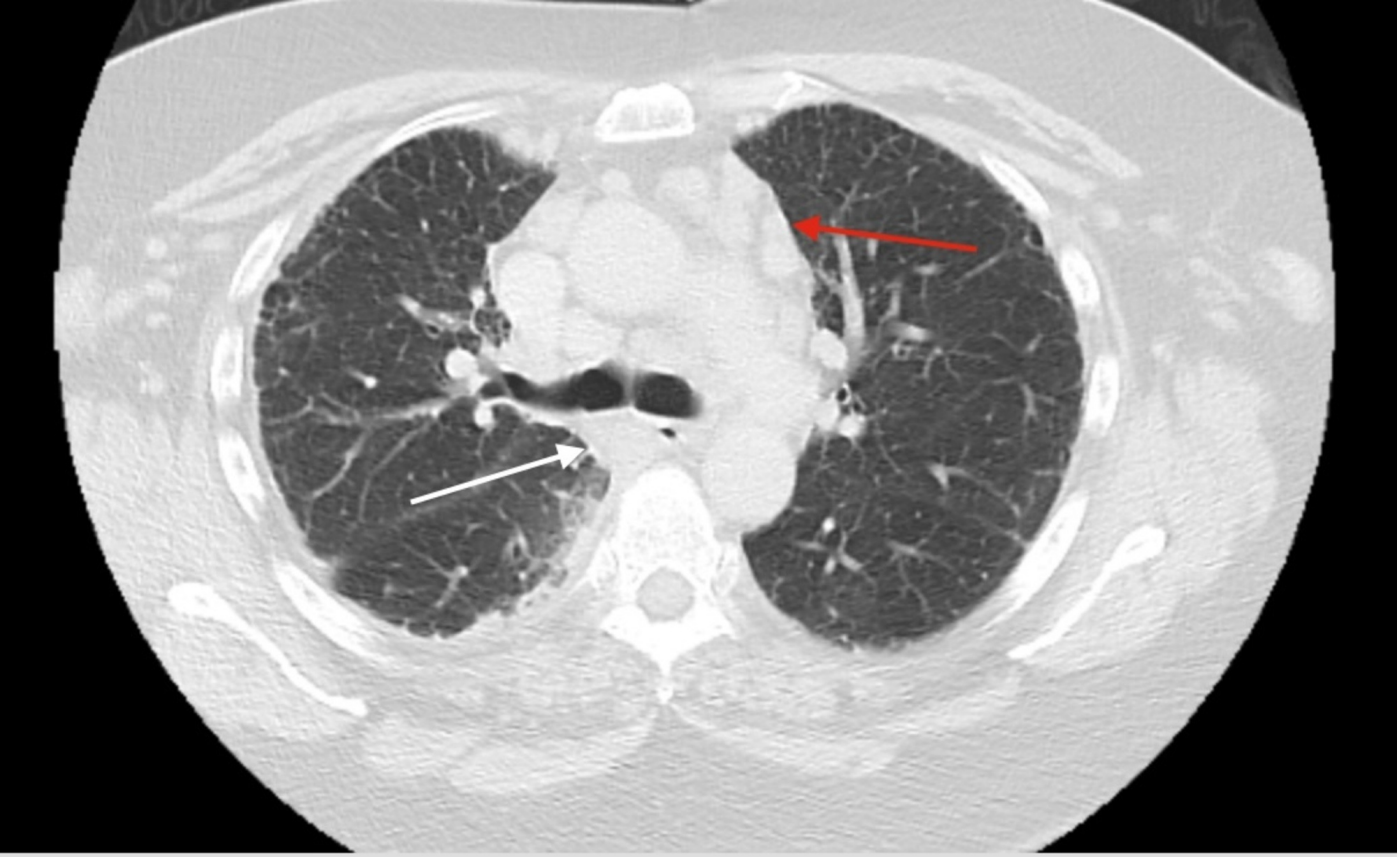
Computed tomography (CT) scan showing enlarged mediastinal and sub-carinal lymph node (see arrows). White arrow: sub-carinal lymphadenopathy Red arrow: mediastinal lymphadenopathy

**Figure 2:**
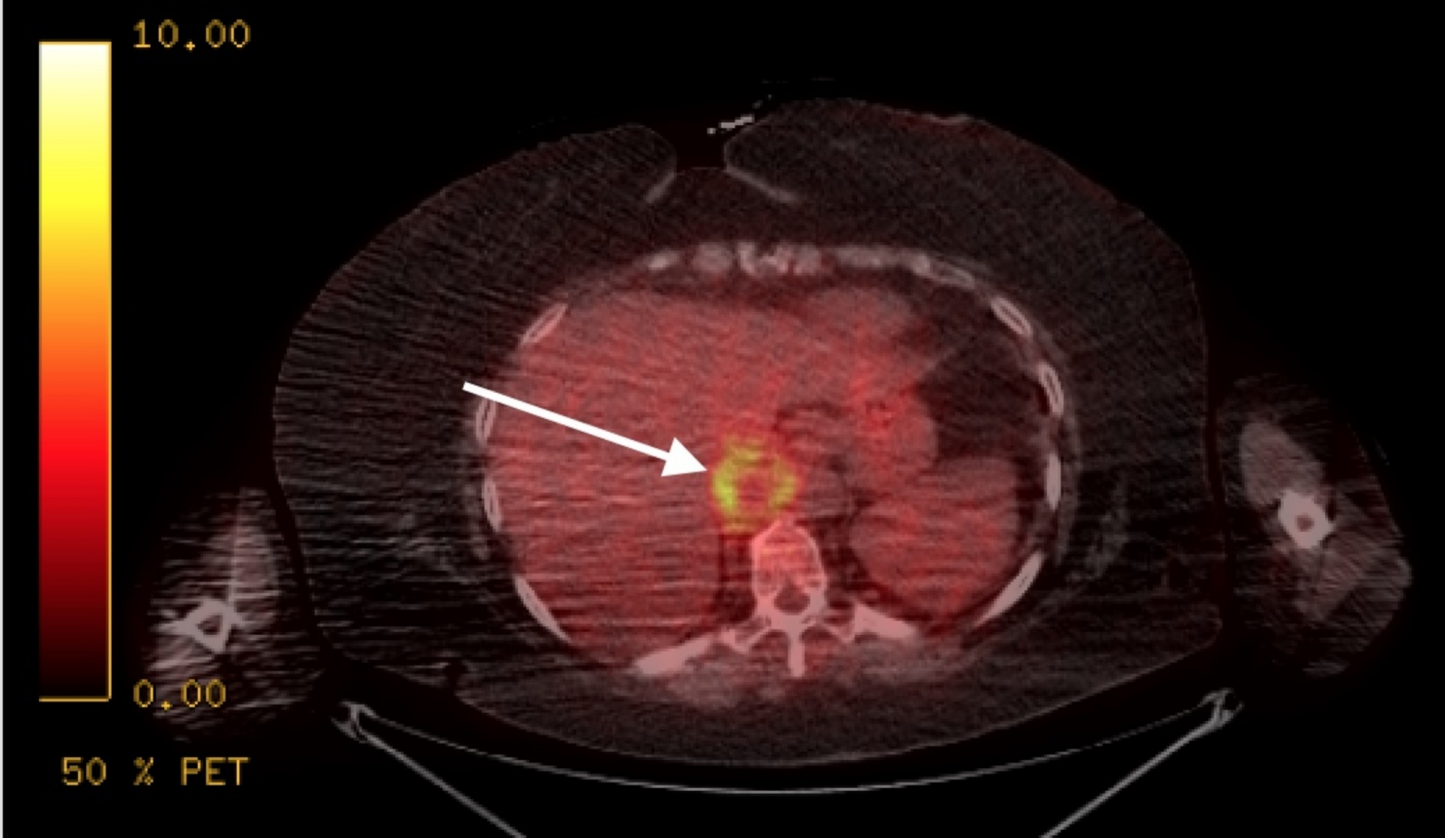
Positron emission tomography-computed tomography (PET-CT) scan of the patient showing a hypermetabolic, enlarged periaortic lymph node to the right of the aorta at the level of the diaphragm (see arrow).

**Figure 3:**
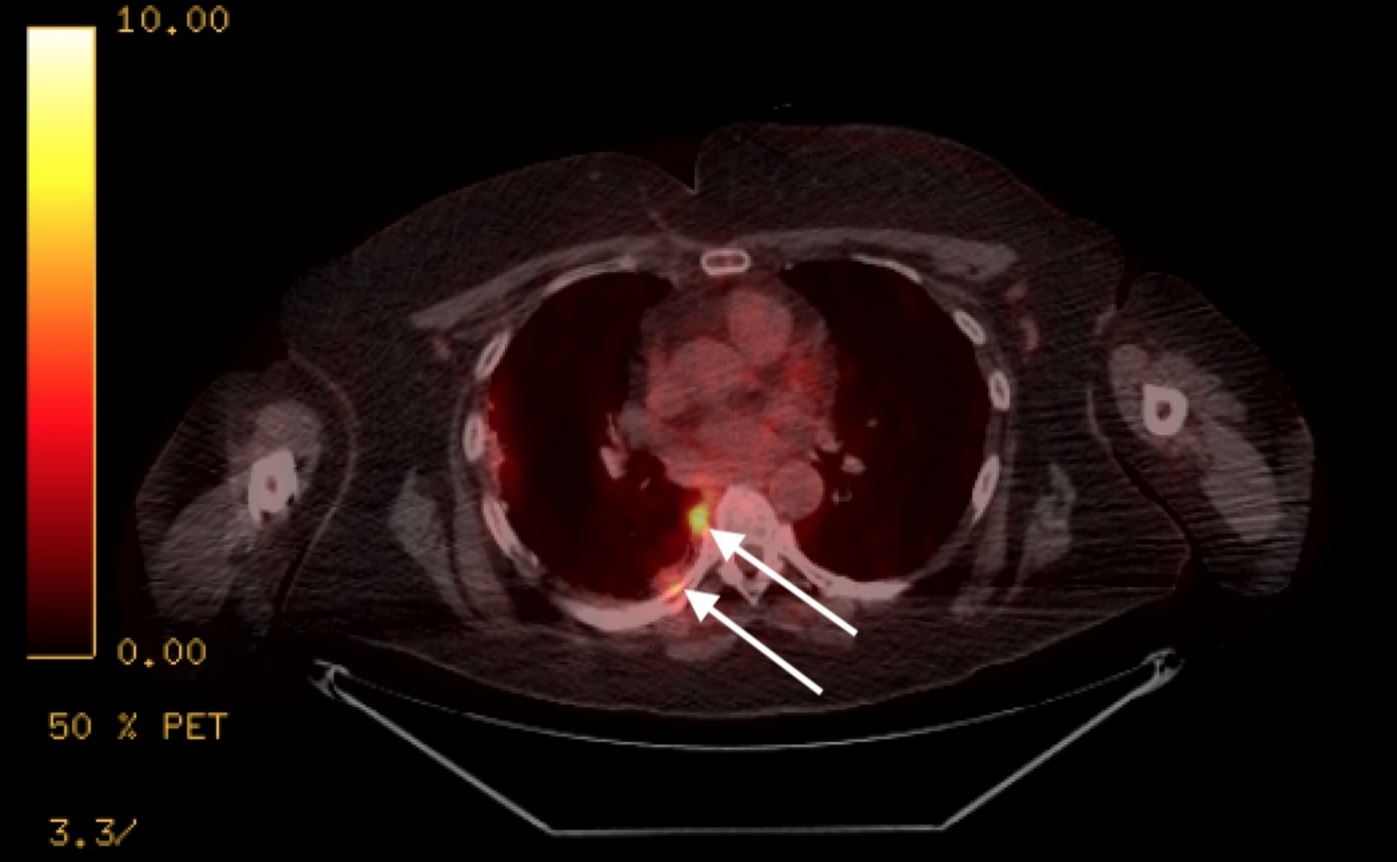
Positron emission tomography-computed tomography (PET/CT) image showing hypermetabolic pleural parenchymal activity along the periphery of the right lung.

**Figure 4:**
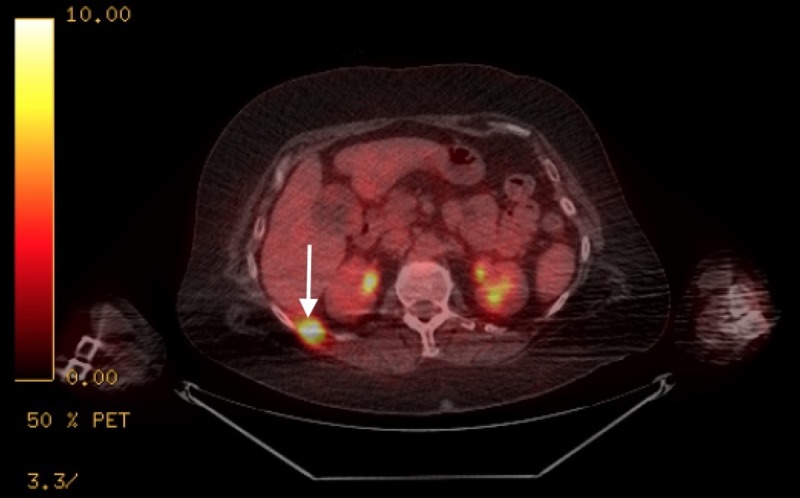
Positron emission tomography-computed tomography (PET/CT) showing area of hypermetabolic activity in the right rib.

**Figure 5:**
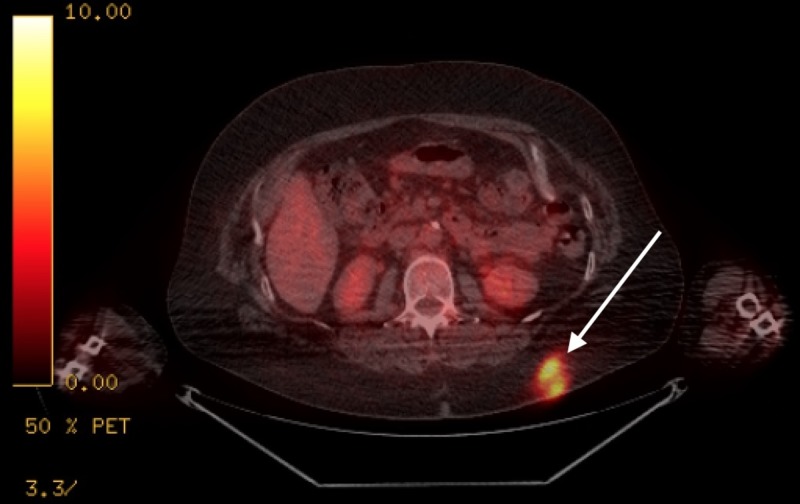
Positron emission tomography-computed tomography (PET/CT) showing hypermetabolic activity in the subcutaneous fat of the upper left buttock area.

Upon presentation to our clinic, the patient had a temperature of 101.8 degrees Fahrenheit, heart rate greater than 90 beats per minute and blood pressure of 86/53, which responded to a one-liter bolus of intravenous fluids. Physical exam was significant for a solid subcutaneous mass measuring 2 x 3 cm. An ultrasound of the mass revealed a 2.4 x 3.0 x 2.8 cm avascular abnormality with lobulated and irregular margins. Subsequent computed tomography (CT) of the abdomen and pelvis divulged two additional masses, one in the right lower rib measuring 36 mm and the other at the diaphragmatic hiatus, which appeared necrotic. A CT of the chest was significant for a patchy area of consolidation in the right lower lobe with several lung nodules measuring up to 1.6 cm in size in both lungs, as well as extensive mediastinal, sub-carinal, hilar and axillary adenopathy (Figure 1). These findings correlated to hypermetabolic areas on a position emission tomography-computed tomography (PET/CT) scan completed a few days prior to her presentation (Figures 2-5). Laboratory data revealed a normocytic anemia (hemoglobin 7.6 g/dL) but otherwise was unremarkable.

She was started on broad-spectrum antibiotics with vancomycin and piperacillin-tazobactam while infectious workup was pending. Ibrutinib was held due to concern for infection. A sputum culture showed the presence of *Aspergillus. *Due to her immune suppression, voriconazole was initiated. Fine needle aspirate was obtained from the rib lesion and subcutaneous lesions. Additionally, bronchoscopy was performed to evaluate the subcarinal lymph nodes and subsequently revealed *Staphylococcus auricularis* on cultures. Flow cytometry from endoscopic biopsy was consistent with her prior diagnosis of CLL, with a monoclonal population of kappa restricted B cells which co-expressed CD5 and CD23. The fungal cultures obtained from the rib, subcarinal lymph node and subcutaneous lesion revealed *Nocardia *species of no specific type. She was transitioned to trimethoprim-sulfamethoxazole, ceftriaxone and imipenem-cilastatin. Immunoglobulin levels were measured, with IgG, IgA and IgM at 1,390, 183 and 18 mg/dL, respectively. Due to IgG level >500 mg/dL, no intravenous immunoglobulin was administered.

The patient then developed a headache and had subsequent magnetic resonance imaging of the brain revealing a solitary enhancing lesion in the anterior limb of the internal capsule with surrounding vasogenic edema. Neurology believed these findings on imaging were likely consistent with CNS involvement from disseminated *Nocardia*. Her hospital course was prolonged and complicated by multiple events, including acute kidney injury and thrombocytopenia felt to be secondary to trimethoprim use. This was discontinued and amikacin was started. She also had development of a transient third-degree heart block and pericardial effusion with resultant pericardiocentesis. Pericardial fluid cultures were without growth and cytology was unremarkable. Subsequently, she developed progressive altered mental status with hypercapnia requiring non-invasive airway support. Workup for etiologies of her altered mental status was non-revealing, including CT of the head. Her clinical status continued to deteriorate. Her family decided to transition her to inpatient hospice care where she died a few days later.

## Discussion

Our case illustrates an unusual presentation with simultaneous infections from two opportunistic pathogens of a patient undergoing treatment for CLL. In the literature, there is only one case of* Nocardia* reported while on ibrutinib [4]. Our patient presented with symptoms concerning for sepsis and infection with fever and abnormal vitals. She had a recent PET/CT scan showing areas of hypermetabolic activity in the rib, subcutaneous tissue as well as lymph nodes. While these areas were initially concerning for malignancy or even Richter's transformation into a high-grade lymphoma due to PET/CT avidity, multiple biopsies were significant for findings of infection and cytology was negative for malignancy. Opportunistic infections are uncommon in patients with CLL and patients are more likely to experience infections from encapsulated organisms such as *Streptococcus pneumoniae* [1]. Due to an excess of clonal B cells, CLL has multiple detrimental effects on both the humoral and cellular immunity due to qualitative and quantitative defects in lymphocytes and neutrophils. CLL alters the ability of neutrophils to fight infections by affecting migration and chemotaxis as well as reduction of their phagocytic and bactericidal activity [1]. Hypogammaglobulinemia is another known consequence of CLL, with patients sometimes requiring intravenous immunoglobulins. Patients often experience poor responses to vaccinations as well. As CLL is an incurable malignancy with an indolent course, treatment is usually only started when the patient becomes symptomatic, such as worsening cytopenias or bulky lymphadenopathy. Treatment may include chemotherapy, chemoimmunotherapy or Bruton tyrosine kinase inhibitors such as ibrutinib [1,5].

Ibrutinib works by inhibition of Bruton tyrosine kinase, which is important in signaling of both the B-cell antigen receptor (BCR) pathway as well as the cytokine receptor pathways that regulate B-cell trafficking, chemotaxis and adhesion [2,3]. The BCR essentially controls the normal proliferation of B-lymphocytes as well as differentiation, apoptosis and other cell processes [2]. Ibrutinib acts to decrease homing of leukocytes [3]. Its role has been extensively studied in multiple hematologic malignancies and is approved for patients with relapsed or refractory CLL and 17p deletion [3]. In the trial which led to its approval, the most frequent adverse effects included diarrhea, fatigue, pyrexia and nausea in the ibrutinib group. Around 24% of patients using ibrutinib experienced grade 3 or higher infections, typically pneumonia and urinary tract infections [6]. However, in a larger systematic review of the literature, over half of patients taking ibrutinib experienced an infection. Around 26% of these patients taking single agent ibrutinib experienced at least a grade 3 infectious event. Grade 5 events of any kind were noted in 10% of patients, with only 2% of those events associated with fatal infections. Most fatal infectious events were secondary to opportunistic infections with the most common being Varicella zoster (n = 15), Aspergillosis (n = 14) and *Pneumocystis jiroveci* (n = 6), followed by cytomegalovirus, *Listeria,* histoplasmosis, *Mycobacterium avium intracellulare* and *Mycobacterium tuberculosis *(n = 1). There was only one case of *Nocardia *infection in our literature review [4].

*Nocardia* typically only causes infection in immunocompromised individuals. It is a gram-positive aerobic bacteria of the order Actinomycetes. *Nocardia *can present as a localized or disseminated infection which may be introduced into the lungs by way of soil, house dust, beach sand, swimming pools and direct inoculation of the skin [7]. There are an estimated 500–1000 cases of *Nocardia *each year, which are typically seen in patients on chronic corticosteroids or immunosuppressants, those with hematologic malignancies or human immunodeficiency virus (HIV) infection [7,8]. In a retrospective study in Spain, Nocardiosis was mostly noted to affect the pulmonary system (77.7%), was disseminated in 18%, and cutaneous in 3.7% [8]. CNS involvement is uncommon but has 100% mortality, followed by disseminated *Nocardia* (64%) and pulmonary Nocardiosis (38.7%) [9].

Our patient presented with concurrent bronchopulmonary *Aspergillus *and disseminated *Nocardia* infection. Upon literature review, the use of ibrutinib in a much larger population has opened the doors for other types of rare opportunistic infections to arise. This is theorized to occur due to its effects on the B-cell signaling pathway, which is an important part of immunity against bacterial infections in particular. This is further demonstrated in patients with congenital BTK mutations who experience higher rates of bacterial infections. Not only is BTK important for various B-cell functions, it is also present in neutrophils, monocytes and macrophages which are important for functional immunity. Additionally, literature suggests that BTK has been implicated as an important contributor to the macrophage response against *Aspergillus* infection in particular, as seen in our case [10].

## Conclusions

This case shows that there is potential for much morbidity and risk of fatal events from the infectious complications related to CLL, both from the disease itself and by mechanisms of treatment. CLL has detrimental effects on the immune system with resultant dysfunction of both humoral and cellular immunity. Additionally, the use of ibrutinib in the treatment of CLL has been associated with the potential for risk of infection, though opportunistic organisms are less commonly noted and also seem to correlate with a higher potential for mortality. Due to this, the potential for infectious complications is a delicate balance in patients with CLL. As two simultaneous opportunistic infections presented in our case, we presume that the use of ibrutinib and the effects of CLL both contributed to an immunocompromised state which unfortunately proved fatal. With the widespread use and tolerability of ibrutinib, there are a growing number of cases with unusual infections such as *Nocardia.* Our case demonstrates the need for increased awareness of potentially fatal opportunistic infections with ibrutinib in order to allow for quick identification and treatment, owing to its high incidence of mortality.
